# Recurrent Pregnancy Loss in Polycystic Ovary Syndrome: Role of Hyperhomocysteinemia and Insulin Resistance

**DOI:** 10.1371/journal.pone.0064446

**Published:** 2013-05-21

**Authors:** Pratip Chakraborty, S. K. Goswami, Shweta Rajani, Sunita Sharma, Syed N. Kabir, Baidyanath Chakravarty, Kuladip Jana

**Affiliations:** 1 Department of Infertility, Institute of Reproductive Medicine, Kolkata, India; 2 Department of Assisted Reproduction, Institute of Reproductive Medicine, Kolkata, India; 3 Reproductive Biology Research, CSIR-Indian Institute of Chemical Biology, Jadavpur, Kolkata, India; 4 Division of Molecular Medicine, Bose Institute, P 1/12, Calcutta Improvement Trust Scheme VIIM, Kolkata, West Bengal, India; Imperial College London, United Kingdom

## Abstract

Recurrent pregnancy loss (RPL) in polycystic ovary syndrome (PCOS), which occurs in ∼50% of total pregnancies is a frequent obstetric complication. Among the several hypotheses, insulin resistance (IR), obesity and hyperhomocysteinemia (HHcy) play significant role/s in RPL. This study was conducted to assess the link between elevated levels of homocysteine and IR in PCOS-associated women with RPL in Kolkata, India. A retrospective study was conducted of one hundred and twenty six PCOS women (<30 years) who experienced two or more spontaneous abortions during the first trimester presenting to Institute of Reproductive Medicine (IRM) in Kolkata during the period of March 2008 through February 2011. One hundred and seventeen non-PCOS subjects with matching age range were randomly chosen as controls. Incidence of HHcy and IR was 70.63% (n = 89) and 56.34% (n = 71), respectively, in RPL-affected PCOS population which was significantly higher (p<0.04; p<0.0001) when compared to the non-PCOS set (HHcy: 57.26%; IR: 6.83%). Rates of miscarriage were significantly higher (p<0.008; p<0.03) in hyperhomocysteinemia-induced miscarriage when compared to the normohomocysteinemic segment (PCOS: 70.63% vs.29.36% & non-PCOS: 57.26% vs. 42.73%) along with the insulin resistant (p<0.04; p<0.0001) population (PCOS: 70.63% vs. 56.34% & non-PCOS: 57.26% vs. 6.83%) in both groups. A probabilistic causal model evaluated HHcy as the strongest plausible factor for diagnosis of RPL. A probability percentage of 43.32% in the cases of HHcy- mediated RPL suggests its increased tendency when compared to IR mediated miscarriage (37.29%), further supported by ROC-AUC (HHcy: 0.778*vs.* IR: 0.601) values. Greater susceptibility towards HHcy may increase the incidence for miscarriage in women in India and highlights the need to combat the condition in RPL control programs in the subcontinent.

## Introduction

Recurrent pregnancy loss (RPL), defined as two or more consecutive pregnancy losses before 20^th^ week of pregnancy is a frequent obstetric complication [1]. While parental chromosomal anomalies, maternal thrombophilic disorders, uterine structural anomalies and anti-phospholipid antibodies have been directly associated with recurrent miscarriage, in almost 50% of cases the pathophysiology remains unknown [2]. Among the undetermined cases, genetic predisposition to venous thrombosis [3] caused by heritable thrombophilic defects and elevation in total homocysteine (tHcy) levels (hyperhomocysteinemia; HHcy) or combinations of these two pathologic conditions have been described as playing a role in the pathogenesis of RPL [4], [5]. Moreover, HHcy may lead to premature vascular disease, i.e., early damage to decidual or chorionic vessels that may cause disturbed implantation of the conceptus [6].However, until now, reports on this topic are actually not univocal [7], [8].

The clinical association of RPL in polycystic ovarian syndrome (PCOS) is more than common. However, the incidence rate between PCOS and recurrent miscarriage remains uncertain due to its wide variation in different studies [9], [10], [11], [12]. The high prevalence of hyper-secretion of LH [13] and obesity [14] in the syndrome contributes to the conclusion which has been reported as a risk factor for spontaneous abortion. Hyperinsulinemia has been proposed as the pathway for the effect of obesity on some reproductive abnormalities, probably through its effect on androgen production. One school of thought has pointed out insulin resistance (IR), an integral pathogenetic feature in the heterogeneous entity as a key factor behind the link between PCOS/obesity and the risk of RPL [15], [16]. Furthermore, a number of studies document a possible association between IR and HHcy [17] with incidence of the latter being increasingly a frequent finding in PCOS women [18]. Recent reports attest the occurrence of hypofibronolysis associated with high plasminogen activator inhibitor -1(PAI-1) in women with PCOS for the reason of RPL [19], [20]. The effects of elevated PAI-1 may also be aggravated by elevated homocysteine [21], eventually causing thrombosis. Furthermore, plasma PAI-1 levels are associated with dyslipidemia, hyperinsulinemia and hypertension which play three essential connecting links in cases with HHcy [22]. Thus, PCOS involves several confounding factors that may contribute, individually or in combination, to thrombosis and eventually lead to RPL. The association of IR, HHcy and obesity in individual with increased miscarriage rates has already been established. However, an effect of IR on HHcy or vice-versa has often been a hindrance to specify the superiority of the any of the variable over the other. In this study, we assessed the relationship between elevated levels of homocysteine and IR noting the effect of BMI in women with RPL, and evaluated the correlation, if any, of these factors with the reproductive outcome in these women.

## Materials and Methods

**Figure 1:**
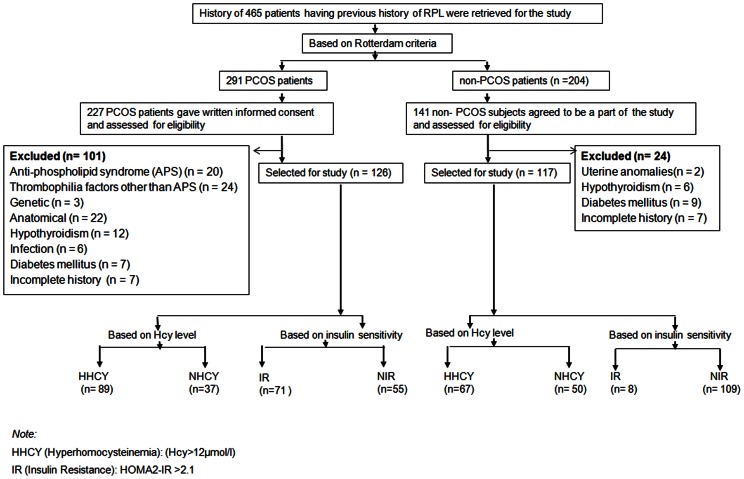
Flow chart for patient's selection characterized by HHcy and/or IR with the incidence of RPL in different sub-groups.

This retrospective study was conducted at Institute of Reproductive Medicine, Kolkata, a referral centre for the treatment and management of infertility and O&G complications. Patients were selected from the women attending the infertility clinic from March 2008 through February 2011. During this period, data of four hundred and sixty five women were approached for the study. Two hundred and ninety one PCOS women as diagnosed in accordance with the Rotterdam criteria [23] and two hundred and four non- PCOS subjects form the total cohort. Two hundred and twenty seven PCOS and one hundred forty one non-PCOS subjects gave written informed consent and were initially enrolled. PCOS was diagnosed if at least two of the following criteria were present: oligo/amenorrhoea, clinical or biochemical hyperandrogenism and PCO on USG. Other common causes of hyperandrogenism (prolactinoma, congenital adrenal hyperplasia, cushing syndrome and virilizing ovarian/adrenal tumors) were excluded. The selection of the patient cohort in the PCOS arm was based on the exclusion criteria: ultrasonography (USG)-documented uterine anatomical anomalies, chromosomal defects as evaluated by peripheral blood karyotyping of both partners, hypothyroidism, diabetes mellitus, positive tests for APS (lupus anticoagulant and anti-cardiolipin antibodies), infections including Toxoplasma gondii, herpes simplex virus, and cytomegalovirus, and unavailability of relevant clinical and laboratory data. One hundred and twenty six women met the inclusion criteria. Twenty-four subjects were excluded from the non-PCOS arm on the basis of uterine anomalies, hypothyroidism and type 2 diabetes mellitus and unavailability of relevant laboratory data. The non-PCOS group of one-hundred and seventeen subjects were represented mostly by those with idiopathic recurrent miscarriage, but also included 3 women who had their miscarriages attributed to male factors. All the three women had infertility due to oligo- or oligoasthenozoospermia. They had their pregnancies by assisted reproductive technology (ART) using husband's semen but experienced loss of pregnancies in all occasions. However, subsequent analysis demonstrated sperm chromosome aneuploidy (>40%) in all the three male partners. They had normal ovulatory cycles (mean ± S.D) of 28±2 days and normal ovarian morphology on ultrasound. (Figure 1). Percentage of pregnancy loss was evaluated with respect to their specific phenotypes. For the present investigation RPL has been defined as two or more spontaneous abortions before 20 weeks of gestation [1]. The body mass index (BMI) was calculated using the Quintelet formula (weight in kg/height in meters squared) and was considered obese if BMI>30 kg/m^2^ according to the World Health Organization [24].

### Ethics statement

The investigation was performed with approval from the Research Ethics Board of the Institute (project no: IRM/IEC/BNC-IHP/37) and informed consent was obtained from all study participants.

### Hormonal and biochemical measurements and calculations

Blood samples were collected between 2^nd^ and 4^th^ days of a menstrual cycle of healthy controls and of women with PCOS after a spontaneous bleeding episode after an overnight fast. TSH, PRL, FSH, LH, testosterone (total), insulin and homocysteine (Hcy) were assayed in serum with automated chemiluminescence assay system using an Immulite® platform (Diagnostic Products Corporation, Los Angeles, CA, USA). The intra- and inter-assay coefficients of variation were <10% for all assays performed. Glucose levels were measured using VITROS dry chemistry system® (Ortho-clinical diagnostics, France). Definition of normal concentrations of plasma homocysteine differs substantially between different studies (5 to 16 µmol/L) although 12 µmol/L were set as the cut-off limit for HHcy. IR was determined using homeostasis model assessment (HOMA2-IR) using the Oxford Diabetes Trials Unit calculator (http://www.dtu.ox.ac.uk; University of Oxford, UK). (HOMA2-IR: (fasting insulin×fasting glucose)/22.5). Patients with HOMA2-IR greater than 2.1 were classified as IR [25]. Outcome of the pregnancy was recorded at the end of the gestational period. Women who did not attend the clinic were contacted by telephone or letter to document information about the outcome of their pregnancy.

### Development of the probabilistic model

**Figure 2:**
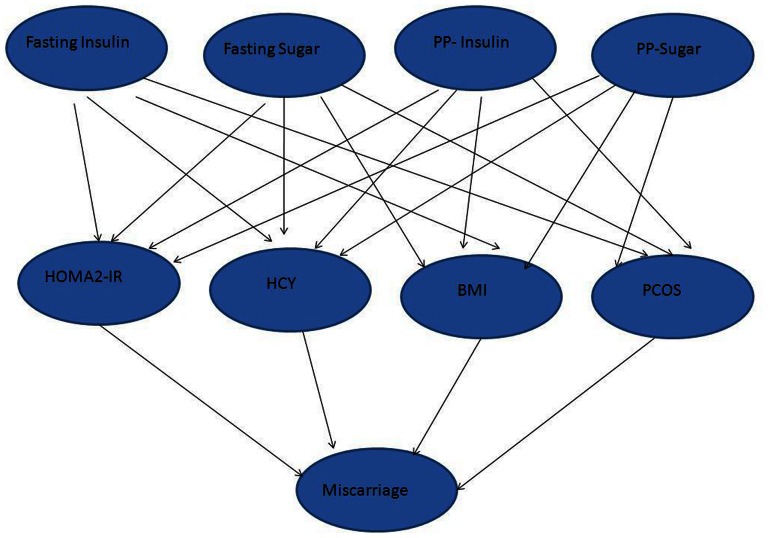
The structure of the model.

A probabilistic model has been developed to compare the variables and their relationships with the help of bayesian network for diagnosis of RPL. The selected database available to us included 243 records where each of the records was described by four (PCOS, BMI, HOMA2-IR and HHcy) different features established as causal factor for miscarriage, which are binary in nature denoting presence or absence of a feature. The model consisted of eight nodes and each node corresponding to a variable namely fasting insulin, post prandial (PP) insulin, PP blood sugar, fasting blood sugar, HOMA2-IR, Hcy, BMI and PCOS. A dependency structure has been developed to correlate the association of IR, HHcy, PCOS and obesity with RPL in single or in combination. Since the above pathologies are influenced by fasting insulin, PP insulin, PP blood sugar and fasting blood sugar, hence all these relationships were incorporated in the model. The structure of the proposed model is shown in Figure 2. After initialization of all conditional probability densities with a uniform distribution, an expectation maximization algorithm was run to estimate the parameters from this given data for the above probabilistic graphical model. After the estimation process is over, we obtained the estimated parameters from the model and computed the probability distribution given by the data.

### Statistical Analyses

The Kolmogorov–Smirnov test was used to test the normality of distribution. All results were expressed as mean ± standard deviation, unless otherwise stated. Differences between the two groups were evaluated with use of analysis of variance and chi-square test, as applicable. Pearson's correlation coefficients were used to calculate correlation between paired data sets.

Statistical Package for Social Sciences (SPSS) (version 17.0, SPSS Inc., Chicago, IL, USA) was used for statistical analysis and SAMIAM software available at http://reasoning.cs.ucla.edu/samiam/ (University of California, Los Angles, U.S.A.) was used for the development of the model. The accuracy of the model was analysed by using a receiver operating characteristic curve (ROC curve) and the area under this curve (AUC). Statistical significance was considered present if the P-value was less than 0.05, unless stated otherwise.

## Results

Anthropometric, biochemical and basal hormonal features of both the groups are summarized in Table 1. Both groups were well-matched in terms of age and marriage duration. However, BMI has been found to be significantly greater (p value<0.01) in PCOS group. Therefore comparisons between women with PCOS and non-PCOS were performed after additional adjustment for BMI. Fasting and post-prandial (PP) insulin was found to be significantly higher (p value<0.0001) in PCOS population along with HOMA2-IR values (PCOS: 2.39±0.91 vs. non-PCOS: 1.51±1.34). Fasting levels of homocysteine (µmol/L) was significantly higher (p value<0.0001) in women with PCOS (PCOS: 13.14±5.04vs. non-PCOS: 8.39±2.22) havingprevioushistory of RPL. (Table 1). Based on characteristic phenotypes, the patients were stratified on the presence or absence of PCOS while subsequent stratification was based on plasma levels of homocysteine (Hcy) and IR.The rate of spontaneous abortion in hyperhomocysteinemic cohort (70.63%) of the PCOS women was significantly higher (p value<0.04) when compared to it's IR-subgroup (56.34%).Moreover, rate of miscarriage between the hyperhomocysteinemic populations of both groups (PCOS:70.63% &non-PCOS: 57.26%), were significantly higher(p value<0.008; p value<0.03) than that of the corresponding normo-homocysteinemic segments (PCOS: 29.36% & non-PCOS: 42.73%) (Figure 1).

**Table 1:** Clinical and biochemical parameters in polycystic ovary syndrome (PCOS) and non-PCOS populations.

Variable	PCOS (N = 126)	Non-PCOS (N = 117)	P- value
Age (years)	28.95±4.28	29.85±3.69	NS
Marriage Duration (years)	5.21±2.60	5.28±2.62	NS
BMI (kg/m^2^)	25.71±3.45	24.79±2.21	<0.01
TSH (µIU/ml)	2.76±1.46	2.49±1.42	NS
Prolactin (ng/ml)	14.21±3.97	13.21±7.79	NS
LH/FSH ratio	1.34±0.71	0.86±0.29	<0.0001
Fasting blood sugar (mg/dl)	89.23±7.53	87.36±7.99	NS
Post-prandial blood sugar (mg/dl)	129.65±29.33	112.24±22.02	<0.0001
Fasting insulin (µIU/ml)	14.39±5.79	7.77±1.21	<0.0001
Post-prandial insulin (µIU/ml)	158.96±70.77	63.28±18.07	<0.0001
HOMA2-IR	2.39±0.91	1.51±1.34	<0.0001
Homocysteine (µmol/L)	13.14±0.61	8.39±2.22	<0.0001

Values are expressed as mean ± S.D. Baseline characteristics of the two groups are evaluated by analysis of variance. Proportions were compared using the chi-square test.

HHcy: hyperhomocysteinemia (Hcy>12 µmol/l); IR: insulin resistance (HOMA2-IR>2.1); HOMA-2-IR: homeostatic model assessment 2- insulin resistance.

A stepwise linear regression analysis demonstrates homocysteine as the independent variable, resulting in a multiple R of 0.317, with a mean square for the regression 158.76, F = 6.805, p value<0.01. Controlling for other variables, HOMA2-IR (r = 0.196, p value<0.029) and PP insulin (r = 0.421, p value<0.001) levels emerged as the most significant parameters affecting homocysteine levels while BMI (r = 0.113, p value<0.21) proved insignificant affecting levels of homocysteine. A significant correlation was noted between homocysteine levels and a history of prior miscarriage (<2) in women with PCOS (r = 0.343, p value<0.05). The correlation maintains significance even after controlling HOMA2-IR values (r = 0.342, p value<0.02) proving HHcy rather than IR to have a stronger association with history of prior miscarriage in these women.

To strengthen our outcome we developed a probabilistic graphical model (Figure 2) with the help of Bayesian network. A most likely diagnosis of 34.37% of unconditional probability has been observed in women experiencing miscarriage. However, the presence of plasma homocysteine increased the probability to 43.32%, while in its absence, the conditional miscarriage probability decreased to 29.03%. No substantial changes in the probability were observed with the presence or absence of PCOS (34.48% and 34.28%) or BMI (35.25% and 34.82%). Nevertheless, the presence of HOMA2-IR increased the probability percentage to 37.29% and the absence of the same reduced the percentage to 31.78%.

**Figure 3:**
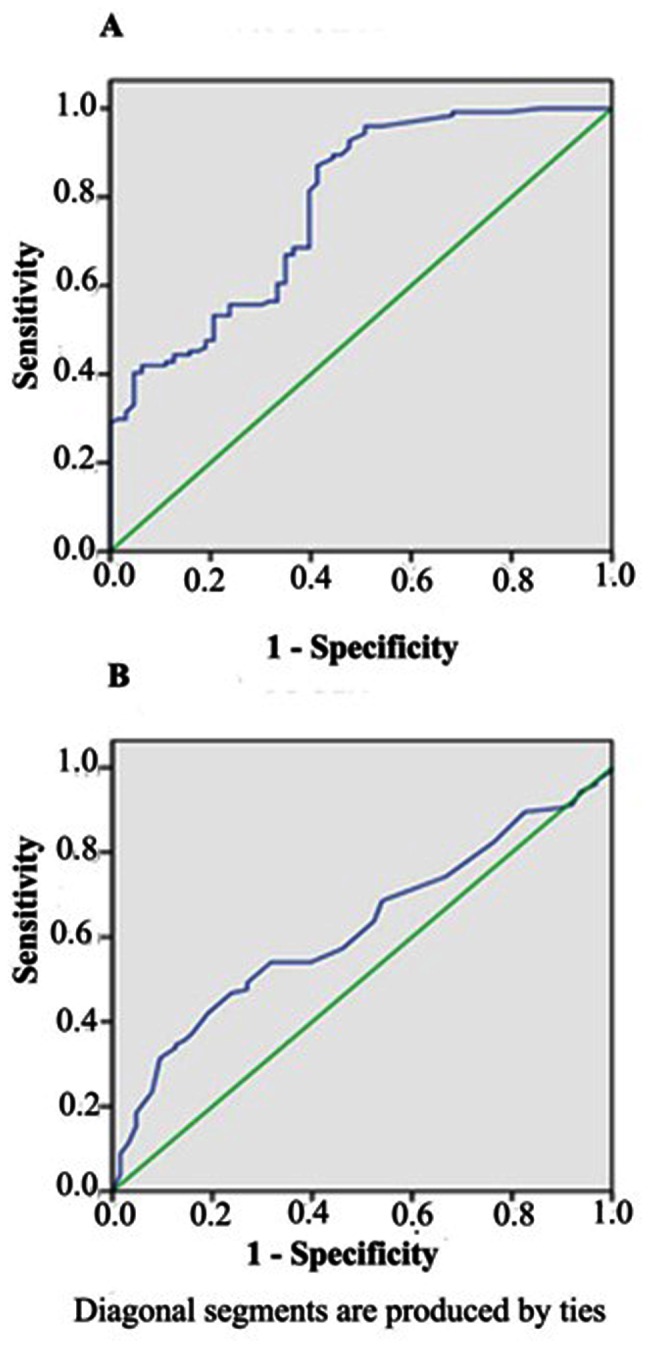
ROC curves of selected variables as a predictor of recurrent pregnancy loss. A: HHcy (AUC = 0.778); B: HOMA2-IR (AUC = 0.619). HHcy = hyperhomocysteinemia; HOMA2-IR = homeostasis model assessment of insulin resistance), AUC = area under the curve.

The accuracy of the model was analysed by using ROC-AUC values. When using a HOMA2-IR cut-off of 2.1the ROC curve had an AUC of 0.619, with a sensitivity of 80.2% and specificity of 62.1%. The predictive accuracy expressed as the ROC-AUC increased slightly when HHcy has been used with a homocysteine threshold of 12 µmol/l, and the ROC curve had an AUC of 0.778, with a sensitivity of 80.7% and a specificity of 70.6%. The individual ROC curves for the HHcy and/or IR in relation to RPL are depicted in Figure 3.

## Discussion

RPL is found in 1–5% of couples desiring pregnancy. Despite various efforts to find an etiologic factor for RPL, ∼50% of these cases remain unexplained. A plethora of factors have been addressed that are associated with the pathophysiology of RPL in PCOS. The common metabolic derailments of the syndrome like obesity and IR have been implicated as individual risk factors for RPL [26]. Recent evidences suggest another metabolic component, HHcy, to be a frequent associate in subjects with PCOS. Moreover, mild-to-moderate HHcy has been suggested as a possible threat to women with habitual abortions or placental abruption [27]. Hence, the heterogeneous syndrome comprises different metabolic factors (IR, obesity, HHcy) which can cause hindrance in isolation or combination for the maintenance of pregnancy. Here we report, HHcy *per se* has got a higher probable association (43.32%) with miscarriage in a small cohort of women diagnosed with RPL.

Two separate populations suffering from RPL were addressed in this investigation: one diagnosed with PCOS, while other was the non-PCOS segment. Both populations were represented with occurrence of HHcy, obesity or IR, which were the phenotypes the further stratifications based upon.

There is no international consensus regarding the establishment of a cut-off point for HHcy and hence different epidemiological studies set up dissimilar cut-off values. However, Hcy values of between 10 and 12 µmol/L in pregnant individuals are considered borderline elevated [28]. Fasting plasma total Hcy value of 12 µmol/L was therefore, set as the cut-off limit for the present investigation. Evaluation of the impact of different degrees of obesity on the risk of recurrent miscarriage demonstrated that not a mild increase in the BMI (25.0–29.9 kg/m^2^), but obesity characterized by BMI>30 kg/m^2^, increases the risk of miscarriage [24]. We therefore set BMI value of 30 kg/m^2^ as the cut-off to differentiate obese from non-obese population.

Rate of miscarriage between the hyperhomocysteinemic populations of both groups were significantly higher than that of the corresponding normo-homocysteinemic segments (p value<0.0001; p value<0.008). Moreover, the subjects in our study showed a significant correlation (r = 0.343, p value<0.05) with the rate of miscarriage in the PCOS population diagnosed according to the Rotterdam criteria which achieved a greater correlation (r = 0.342, p value<0.02) after controlling for the HOMA2-IR values. This suggests that HHcy may set the ground behind the risk of RPL irrespective of IR/non-IR subjects. Alongside, it also raises the issue concerning earlier studies that whether HHcy was segregated from the insulin resistant population to envisage its individual role to RPL.

In the current investigation, a linear regression analysis described homocysteine as the independent variable affecting RPL. However, the connection between IR and HHcy stands tall as HOMA2-IR (r = 0.196, p value<0.029) and PP insulin (r = 0.421, p value<0.001) levels emerged as the most significant parameters after controlling for other variables, affecting homocysteine levels with no significant correlation with BMI. We however didn't take interest to evaluate miscarriage owing to obesity as a mild increase in the BMI (25.0–29.9 kg/m^2^) found in our patient cohort do not really impact on the risk of recurrent miscarriage [24].

Bayesian networks have been around in biomedicine and health-care for more than a decade and have become increasingly popular for handling the uncertain knowledge involved in establishing diagnoses of disease. For controlling the diagnostic complexity in the field of RPL, a proper understanding of the processes is important as is the ability to reason about them. It is apparent that the numbers of variables are many and to come to a conclusion as to which variable(s) is/are playing leading part, network analysis definitely helps under such circumstances. From the learned model, a most likely diagnosis of 34.37% of unconditional probability has been observed in women experiencing miscarriage. While using a homocysteine threshold of 12 µmol/L, HOMA2-IR: 2.1, BMI: 30 kg/m^2^ and on the presence or absence of PCOS, we found that HHcy has got the maximum probable associate ship with the rate of miscarriage in women in Kolkata, India. This was supported by the ROC-AUC predictive accuracy expressed, as it increased slightly to 0.778 while using a homocysteine threshold of 12 µmol/L.

A high proportion of PCOS patients have a greater risk for spontaneous abortion due to the individual anatomic defect or the confounding factors associated with it [26]. The comparison among PCOS and non-PCOS patients in the current study gave us opportunity to cue at the anatomical problem alone. However, we did not find any significant associate-ship of PCO morphology in the designed probabilistic model.

The mechanism linking IR with RPL may be complicated. Hyperinsulinemia adversely affects the pre-implantation environment by decreasing the expression of glycodelin and IGF-binding protein-1 [29] which may play a role in inhibiting the endometrial immune response of the embryo, and seems to facilitate adhesion processes at the feto-maternal interface. A positive relationship between HOMA2-IR and spontaneous abortion in quite a few numbers of studies suggest IR as a significant predictor of pregnancy wastage [15], [16]. Elevated PAI-I (an endogenous inhibitor of fibrinolysis) levels have an independent association-ship with recurrent miscarriages in women in this respect [21]. A recent report documented in patients receiving metformin that PAI-1is positively correlated with the HOMA score [30]. In the small patient cohort selected, HOMA2-IR stands as the second most important individual causal factor to be linked with the risk of RPL as analysed by the ROC curve and the networking model.

Increased thrombosis caused by HHcy resulting microthrombi formation in the vessel bed of the placenta can impair sustained placental function [3]. These microthrombi may cause multiple placental infarctions and subsequently maternal complications of pregnancy. Apart from the thrombogenic effect of elevated Hcy on pregnancy in women with PCOS, few recent studies have also implicated the adverse effect of high serum or follicular fluid Hcy levels on defect in folliculogenesis [31], embryo quality [32], oocyte numbers and oocyte maturation [33], that may have future bearings on the establishment and maintenance of pregnancy. Thus, homocysteine may be responsible for more reasons than one for the increased miscarriage rates.

The current study has some limitations. The gold standard for establishing IR is euglycemic-hyperinsulinemic clamp which has not been in routine clinical practice. Instead, we used HOMA2-IR calculation which is often used as a surrogate marker for IR [34]. Along with this, the variables by which homocysteine levels are influenced like smoking, renal function, vitamin B status and enzyme dysfunction states were not examined in this study. However, all of the patients in our clinic are actively discouraged from smoking.

In conclusion, HHcy mediated RPL is one of the most significant contributor which plays a substantial role in spontaneous abortion of sub-continental women. Moreover, various studies report the role of folic acid [35], [36], Vit B6 & B12 supplementation [37] and LMWH (unpublished observations) in improving pregnancy outcomes in women with HHcy. Thus, the role of identification and active treatment of Hcy in these women at high risk of subsequent miscarriage should be emphasised upon. Further studies are required to confirm our findings and to assess the efficacy of vitamin supplementation &/or anticoagulation in prevention of further miscarriage in women with HHcy.
